# O027: Compliance of jordanian registered nurses with infection control guidelines: a national population-based study

**DOI:** 10.1186/2047-2994-2-S1-O27

**Published:** 2013-06-20

**Authors:** OM Al-Rawajfah, IM Hweidi, M Alkhalaileh, YS Khader, SA Alshboul

**Affiliations:** 1 Adult health nursing, Al al-Bayt University/ Faculty of Nursing, Mafraq, Jordan; 2 Adult health nursing, Jordan; 3 Department of Public Health, Community, Family Medicine/Faculty of Medicine, Jordan university of Science and Technology, Irbid, Jordan; 4 Faculty of Faculty of Applied Medical Sciences, Jordan university of Science and Technology, Irbid, Jordan

## Introduction

The CDC recommends that educating health-care workers regarding infection control measures is the first high priority strategy to prevent and control Health Care Related Infection (HCRIs). The CDC recommends periodic assessment of knowledge and adherence to infection control guidelines as a high priority. Such guidelines are updated and published frequently.

## Objective

This national study aims to assess the compliance of Jordanian RNs with standard Infection Control (IC) guidelines.

## Method

Cross-sectional, descriptive design was used. Proportional-multistage, probability sampling was used to obtain a sample of 10% of all staff nurses working in Jordanian hospitals. The final sampling frame consisted of 103 hospitals from different healthcare sectors. Standardized self-reported instruments used to evaluate the compliance. In the current study the reliability coefficient of the tool was 0.88. Ethical approval was obtained from participating hospitals. Informed consent was obtained from participating nurses.

## Results

A 889 RN from 22 hospitals were participated in the study with a response rate of 89.4%, of which 52.6% were females, 81.9% were holding a Bachelor degree. The mean age was 29.0 years (*SD* = 5.9) and the mean years of experience was 6.9 years (*SD* = 5.8). The overall mean compliance score was 119.9 (*SD* = 14.3). Nurses who received IC training in the hospital demonstrated higher compliance (*M* = 120.2, *SD* = 13.6) than those who never received such training (*M* = 115.8, *SD* = 15.2), *p* < 0.001. Nurses who work in university affiliated hospitals demonstrated higher compliance than other types of hospital (*p* < 0.001).

## Conclusion

This study demonstrated the necessity of establishing need-based IC programs especially for newly employed nurses. This study provides information about infection control practices in various healthcare sectors in Jordan. Results from this study expected to guide efforts to develop educational tools, programs, and curricula to improve infection control practices in the Jordan.

## Disclosure of interest

O. Al-Rawajfah Employee of no conflicts of interest, Grant/Research support from no conflicts of interest, I. Hweidi Employee of no conflicts of interest, Grant/Research support from no conflicts of interest, M. Alkhalaileh Employee of no conflicts of interest, Grant/Research support from no conflicts of interest, Y. Khader Employee of no conflicts of interest, Grant/Research support from no conflicts of interest, S. Alshboul Employee of no conflicts of interest, Grant/Research support from no conflicts of interest.

